# Influence of helicobacter pylori infection on Chinese adult males’ body muscle mass: a cross-sectional and cohort analysis

**DOI:** 10.3389/fcimb.2025.1575108

**Published:** 2025-05-29

**Authors:** Ju-Hua Liu, Yun Li, Rui-Ning Nie, Jun-Xiang Li, Fang-Yuan Cheng, Yu-Xin Su, Yun-Feng Yang, Jian-Wei Gu

**Affiliations:** ^1^ Department of Geriatrics, Affiliated Hospital of North Sichuan Medical College, Nanchong, Sichuan, China; ^2^ Health Management Center of Affiliated Hospital of North Sichuan Medical College, Nanchong, Sichuan, China

**Keywords:** helicobacter pylori, bioelectrical impedance analysis, body muscle mass, adult Chinese males, a cross-sectional study, cohort analysis

## Abstract

**Aim:**

Helicobacter pylori (HP) infection may cause many disorders outside the digestive system, although research on the association between HP infection and muscular atrophy among males is limited. This study aimed to examine the influence of HP infection on adult Chinese males’ body muscle mass using a cross-sectional and cohort analysis.

**Methods:**

A total of 8110 Chinese adult males were enrolled in the study. HP infection was assessed using the C13 breath test. Using bioelectrical impedance analysis (BIA), body muscle mass was detected and total muscle mass, total skeletal muscle mass, and appendicular skeletal muscle mass index (ASMI) were recorded. Univariate and multivariate linear regression analyses were conducted to identify the factors associated with body muscle mass.

**Results:**

Subjects with HP infection had a lower total muscle mass, total skeletal muscle mass, and ASMI compared with those without HP infection (P < 0.01). Univariate and multivariate linear regression analyses demonstrated that HP infection was an independent risk factor of total muscle mass, total skeletal muscle mass, and ASMI (P < 0.01); this association persisted when subjects were divided into young, middle-aged, and elderly according to age (P < 0.01). A subsequent cohort study confirmed that persistent HP infection accelerated the pathological process of muscle decline (P < 0.01).

**Conclusion:**

HP infection is an independent risk factor for muscle decline in adult Chinese males, long-term HP infection may accelerate this pathological progression.

## 1 Introduction

The muscles of the human body, including skeletal, cardiac, and smooth muscles, are responsible for various physiological functions, such as metabolism, movement, and postural stability. Skeletal muscles, which are attached to bones, are primarily responsible for physical activity, maintaining posture, protecting internal organs, and serving as the main tissue for energy metabolism. The appendicular skeletal muscle mass index (ASMI), an indicator used to assess individual muscle mass by standardizing the mass of skeletal muscles in the limbs, is widely used in health assessments for subjects with chronic diseases, especially sarcopenia (Ji et al., 2023; Chen DQ. et al., 2024). Factors such as aging, genetics, malnutrition, chronic disease, and inflammation conditions (Webster et al., 2020; Wang et al., 2021; Naruse et al., 2023; Zhu et al., 2024) can accelerate the pathological process of muscle decline, leading to muscular atrophy, which is associated with various adverse outcomes such as physical disability, impaired cardiopulmonary function, unfavorable metabolic effects, falls, poorer quality of life, and death (Nguyen et al., 2024). Human skeletal muscle undergoes inevitable structural and physiological changes with aging, associated with a 3-8% decrease per decade after the age of 50 (Gao et al., 2025). This is mainly due to age-related metabolic resistance (such as decreased IGF-1 levels) (Ahmad et al., 2020), mitochondrial dysfunction (Dulac et al., 2021), chronic low-grade inflammation (Coperchini et al., 2025), and degeneration of the neuromuscular junction (Motanova et al., 2024).Currently, there is no effective treatment for muscular atrophy; therefore, identifying risk factors that can cause muscular atrophy at an early stage and taking effective measures are crucial in preventing the occurrence of adverse events.

Helicobacter pylori (HP), a Gram-negative bacillus that is widespread all over the world and infects more than 50% of the world’s population, may cause many disorders of the upper gastrointestinal tract by invading the gastric mucosa (Verma et al., 2024). Numerous studies indicate that HP infection is also associated with various non-gastrointestinal disorders through chronic inflammatory responses, nutritional status, and metabolic disorders (Krupa et al., 2021; Tang et al., 2024). Studies have indicated that HP infection is an independent risk factor for low skeletal muscle mass in middle-aged females and that HP eradication can reduce the risk of low muscle mass in elderly women [ (Baeg et al., 2015; Xu et al., 2023). However, there is limited research on whether there is an association between HP infection and muscle mass in adult males. Thus, the objective of this study was to conduct a large-scale cross-sectional and cohort analysis to investigate the effects of HP infection on muscle mass in adult Chinese males.

## 2 Materials and methods

### 2.1 Study population

The study comprised subjects who underwent routine health examinations at the Department of Health Management Center, Affiliated Hospital of North Sichuan Medical College, Sichuan Province, China, from January 2021 to December 2024. The inclusion criteria were males who 1) were above 18 years of age, 2) had complete personal information and blood biochemical results, and 3) completed the C13 urea breath test and bioelectrical impedance analysis (BIA). The clinical parameters included age, height (kg), and weight (m), and body mass index (BMI) was calculated in kg/m2. Blood pressure was measured after the subjects rested for at least 5 min. The serum inflammatory parameters included high-sensitivity C protein (hsCRP), number of white blood cells (WBC), lymphocytes, platelets, neutrophils, and monocytes. A neutrophil-to-lymphocyte ratio (NLR), monocyte-to-lymphocyte ratio (MLR), or platelet-to-lymphocyte ratio (PLR) was obtained by dividing the number of neutrophils, monocytes, or platelets by the number of lymphocytes. NMR was obtained by dividing the number of neutrophils by the number of monocytes. The nutritional status of the study subjects was reflected by their serum levels of albumin, total protein, and hemoglobin. Metabolic indexes included fasting blood glucose (FBG), total cholesterol (TC), triglyceride (TG), high-density lipoprotein cholesterol (HDL), low-density lipoprotein cholesterol (LDL), serum levels of creatinine, and uric acid. We excluded subjects who could not independently cooperate to complete BIA analysis, underwent gastric resection (including partial or total resection), and had severe heart failure (NYHA class III or above), severe liver or kidney failure, malignant tumors (currently in the active phase or recently treated), limb deficiencies, or been immobilized for a long period due to injury or illness in the past three months. A total of 8,110 adult males were enrolled in the study; moreover, the study included 426 individuals who underwent at least two medical examinations, with a time interval of more than 1 year between the initial and final check-up, did not receive treatment for HP eradication, and maintained a positive or negative HP status throughout the follow-up period. The study was approved by the Ethics Committee of the Affiliated Hospital of North Sichuan Medical College, and all procedures performed in the study were in accordance with the ethical standards of the institutional research committee and with the 1964 Helsinki Declaration and its later amendments.

### 2.2 Detection of HP infection

HP infection was detected using the C13 breath test (Yang et al., 2020), which was conducted on an empty stomach or 2 h after a meal. The inspection method of the test was as follows: The HY-IREXB carbon 13 exhalation detector (Huayou Mingkang Photoelectric Technology, China) was used to measure the DOB value before and after the urea [13C] granules were taken orally. HP infection was defined as positive if the difference in the DOB value between the two measurements was > 4.

### 2.3 Detection of body muscle mass

Body muscle mass was assessed using a BIA device (BIA 101 RJL, Donghua, Guangzhou, China), which is a non-invasive and cost-effective method for evaluating body composition and is acknowledged by international guidelines as a valid alternative to whole-body dual-energy DXA in large-scale studies (Kyle et al., 2004). According to the guidelines, with a room temperature ranging from 22–25°C, during BIA measurements, Subjects were instructed to first urinate, then fast for 8 hours, followed by resting for 5 to 10 minutes. After resting, they were asked to remove their shoes and socks and stand barefoot on the pedal, aligning their heels with electrodes at the rear end of the foot. The subjects were then asked to hold the electrodes with both hands, keeping their arms relaxed at the sides without touching their body, and ensuring good contact between the electrodes on their hands and feet and the device electrodes. The device conducted low-intensity electrical currents and recorded the impedance values of various body components, including total muscle mass and total skeletal muscle mass. The final results were automatically displayed by the device based on the subject’s gender, age, height, and other basic data. ASMI was calculated by the ratio of the total skeletal muscle mass of the limbs to the square of height – ASMI = [total skeletal muscle mass of limbs (kg)/height² (m²)].

### 2.4 Statistical analysis

Data are expressed as mean ± standard deviation for continuous variables and percentage for categorical variables. Independent student’s t-test, chi-squared test, and Mann–Whitney’s U-test were conducted to assess the statistical significance between subjects with and without HP infection. A paired student’s t-test was used to assess the longitudinal association between HP infection status and different types of muscle mass. Multivariate linear regression analyses were performed to investigate the effect of HP infection on muscle mass by adjusting for various risk factors with P < 0.05 in the univariate linear regression analysis. Collinearity statistics were used to assess the collinearity of the parameters used in the multivariate models; values greater than 10 for VIF and less than 0.1 for tolerance were considered to require action. Statistical analyses were performed using SPSS (version 22.7) for Windows, with P < 0.05 considered statistically significant.

## 3 Results

### 3.1 Clinical characteristics

The eligible subjects had a mean age of 46.31 ± 11.78 years, ranging from 18 to 89 years. The prevalence of HP infection in the subjects was 43.92% (3,562). **Table 1** presents the clinical characteristics of subjects with and without HP infection. The HP+ group was older and had a lower BMI and blood pressure value compared with the HP-group (P < 0.05). According to the inflammatory indicators, the HP+ group had higher hs-CRP, NLR, NMR, and MLR compared with the HP- group (P < 0.01). With regards to nutrition index, serum levels of albumin, total protein, and hemoglobin were lower in the HP+ group compared with that of the HP- group (P < 0.01). Although the serum levels of albumin and total protein levels were all above the normal range (above 35g/l and 63g/l) in the present study, after dividing the study subjects into quartile groups based on serum albumin levels (47.0(45.2, 48.4) g/l) and total protein levels (75.9(73.3, 78.5) g/l) respectively, the HP+ group had the highest number of individuals in the Q1 level (28.4% and 27.1% respectively), and the lowest number of individuals in the Q4 level (22.5% and 23.9% respectively); whereas the HP- group had the lowest percentage in Q1 levels (23.4% and 24.6% respectively) but the highest percentage in the Q4 group (26.7% and 26.8% respectively). A total of 639 subjects with hemoglobin levels below 130g/l, 69.4% of whom were with HP infection. Referring to metabolic indicators, the HP+ group exhibited higher levels of HDL-c but lower levels of TG, LDL-c, creatinine, and uric acid compared to the HP- group (P < 0.05). There were no significant differences in WBC, PLR, FBG, and TC between the two groups (P > 0.05).

**Table 1:** Clinical characteristics between subjects with and without helicobacter pylori infection.

Variable	Total (n=8110)	HP-(n=4548)	HP+(n=3562)	P
Clinical database				
Age (years)	46.31 ± 11.78	45.82 ± 11.78	46.93 ± 11.77	<0.01
BMI (kg/m^2^)	25.67 ± 3.20	26.18 ± 3.02	25.01 ± 3.30	<0.01
SBP (mmHg)	126.60 ± 16.41	127.28 ± 15.67	125.75 ± 17.27	<0.01
DBP (mmHg)	78.60 ± 11.71	79.23 ± 11.34	77.80 ± 12.10	<0.01
Plasmea inflammatory markers				
WBC (10^9/L)	6.35 ± 1.58	6.34 ± 1.58	6.36 ± 1.58	0.45
hs-CRP (mg/L)	0.61(0.25-1.48)	0.55(0.23-1.27)	0.67(0.28-1.68)	<0.01
NLR ratio	2.01 ± 0.87	1.99 ± 0.84	2.04 ± 0.91	0.02
PLR ratio	110.25 ± 37.82	110.37 ± 36.92	110.11 ± 38.94	0.77
MLR ratio	0.19 ± 0.05	0.18 ± 0.06	0.19 ± 0.06	<0.01
NMR ratio	5.85 ± 1.84	5.78 ± 1.82	5.94 ± 1.87	<0.01
Nutrition-related index				
Albumin (g/L)	47.00 ± 2.62	47.17 ± 2.60	46.78 ± 2.63	<0.01
Total protein (g/)	75.91 ± 3.99	76.04 ± 3.97	75.74 ± 4.01	<0.01
Hemoglobin (g/)	15.60 ± 14.61	154.19 ± 12.77	148.30 ± 16.08	<0.01
Metabolic indexes				
FBG (mmol/L)	5.30 ± 1.58	5.29 ± 1.48	5.30 ± 1.70	0.80
TC (mmol/L)	5.02 ± 1.95	5.03 ± 0.95	5.02 ± 0.95	0.44
TG (mmol/L)	1.92 ± 1.05	2.01 ± 1.59	1.81 ± 1.59	<0.01
LDL-C (mmol/L)	2.94 ± 0.80	2.97 ± 0.79	2.91 ± 0.80	<0.01
HDL-C(mmol/L)	1.22 ± 0.30	1.18 ± 0.27	1.27 ± 0.33	<0.01
Creatinine (umol/L)	79.19 ± 14.30	77.24 ± 12.86	72.57 ± 15.57	<0.01
Uric acid (umol/L)	384.65 ± 94.39	399.64 ± 89.90	365.50 ± 96.52	<0.01

BMI, body mass index; DBP, diastolic blood pressure; FBG, fast blood glucose; HDL-C, high-density lipoprotein cholesterol; hs-CRP, high sensitivity C protein; LDL-C, low-density lipoprotein cholestero; MLR, monocyte-to-lymphocyte ratio; NLR, neutrophil-to-lymphocyte ratio; NMR, neutrophil-to-monocyte ratio; NRI, Nutritional Risk Index; PLR, platelet-to-lymphocyte ratio; TC, total cholesterol; TG, triglyceride; WBC, white blood cell.

### 3.2 Comparison of different types of muscle mass in subjects with and without HP infection

**Figure 1:**
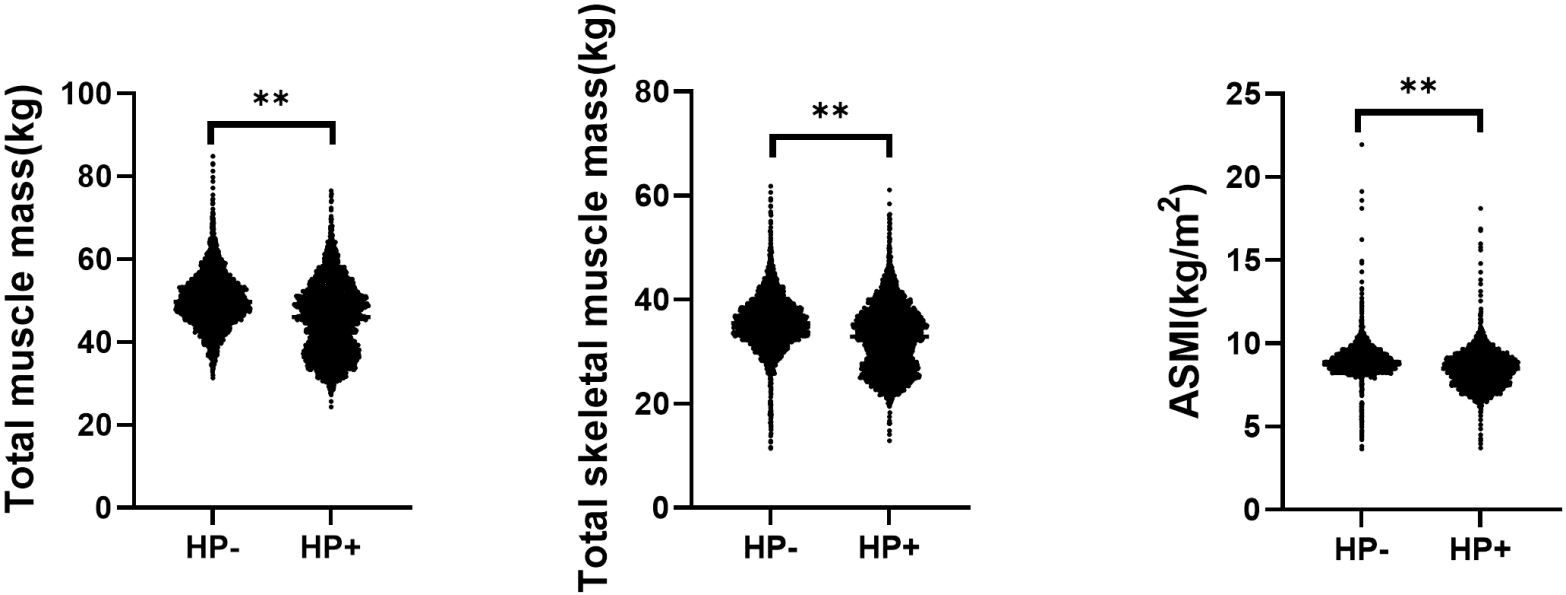
Comparison of different types of muscle mass in subjects with and without helicobacter pylori infection. ASMI, Appendicular skeletal muscle mass index; HP, Helicobacter pylori. ^**^<0.01.

The comparison of different types of muscle mass in subjects with and without HP infection is shown in **Figure 1**. Both total muscle mass (50.10 ± 6.41 vs. 45.64 ± 8.43 kg, P < 0.01), total skeletal muscle mass (35.81 ± 8.94 vs. 32.66 ± 7.09 kg, P < 0.01), and ASMI (8.98 ± 2.02 vs. 8.48 ± 1.14 kg/m^2^, P < 0.01) were all higher in the HP- group compared with that of the HP+ group.

### 3.3 Influence of HP infection on total muscle mass

Based on the results of univariate linear regression analysis (**Table 2**), age, BMI, blood pressure, serum inflammatory markers, and nutrition-related parameters (excluding total protein), as well as metabolic indicators (excluding TC), were found to be associated with total muscle mass (P < 0.01). The VIF for parameters with P < 0.05 in univariate linear regression was below 10, and the tolerance for these parameters was above 0.1, indicating no significant collinearity among them. Consequently, all these variables were included in the multivariate linear regression analysis, and HP infection remained an independent factor associated with total muscle mass (β = -1.75, 95% CI = -2.10 to -1.39, P < 0.01).

**Table 2:** Influence of helicobacter pylori infection infection on total muscle mass.

Variables	Univariate analyse		Multivariate analyse	
	β (95%CI)	P	β (95%CI)	P
HP+	-4.46 (-4.78–4.14)	<0.01	-1.75 (-2.10–1.39)	**<0.01**
Clinical database				
Age (years)	-0.15 (-0.17–0.14)	<0.01	-0.14 (-0.16–0.12)	**<0.01**
BMI (kg/m^2^)	1.21 (1.17-1.26)	<0.01	0.85 (0.79-0.91)	**<0.01**
SBP (mmHg)	0.06 (0.05-0.07)	<0.01	-0.01 (-0.03-0.01)	0.32
DBP (mmHg)	0.11 (0.10-0.13)	<0.01	0.0 (-0.01–0.04)	0.31
Serum inflammatory markers				
WBC (10^9/L)	0.63 (0.53-0.74)	<0.01	-0.08 (-0.23-0.07)	0.28
hs-CRP (mg/L)	0.26 (0.16-0.36)	<0.01	-0.7 (-0.15-0.01)	0.08
NLR ratio	-0.61 (-0.80–0.41)	<0.01	-0.64 (-0.95–0.33)	**<0.01**
PLR ratio	-0.02 (-0.03–0.02)	<0.01	-0.01 (-0.01-0.00)	**0.04**
MLR ratio	4.85 (2.08-7.62)	<0.01	7.87 (1.21-14.53)	**0.02**
NMR ratio	-0.29 (-0.39—0.20)	<0.01	-0.21 (-0.41–0.01)	**0.03**
Nutrition-related parameters				
Albumin (g/L)	0.41 (0.35-0.47)	<0.01	0.02 (-0.05-0.09)	0.55
Total protein (g/L)	-0.03 (-0.07-0.02)	0.23		
Hemoglobin (g/L)	0.25 (0.24-0.26)	<0.01	0.12 (0.11-0.13)	**<0.01**
Metabolic indexes				
FBG (mmol/L)	0.16 (0.05-0.27)	<0.01	0.18 (0.07-0.29)	**<0.01**
TC (mmol/L)	0.06 (-0.12-0.24)	0.51		
TG (mmol/L)	1.05 (0.95-1.15)	<0.01	0.11 (–0.03-0.25)	0.11
LDL-C (mmol/L)	0.58 (0.36-0.79)	<0.01	-0.50 (-0733–0.27)	**<0.01**
HDL-C (mmol/L)	-9.85 (-10.36–9.34)	<0.01	-1.40 (-2.06–0.74)	**<0.01**
Creatinine (umol/L)	0.24 (0.23-0.25)	<0.01	0.15 (0.13-0.16)	**<0.01**
Uric acid (umol/L)	0.04 (0.03-0.04)	<0.01	0.01 (0.00-0.01)	**<0.01**

Similar to **Table 1**; HP, Helicobacter pylori.

The bolded values represent statistically significant results.

### 3.4 Influence of HP infection on total skeletal muscle mass

Similar to the results of total muscle mass, age, BMI, blood pressure, serum inflammatory markers, nutrition-related parameters excluding total protein, and metabolic markers excluding TC were all associated with total skeletal muscle mass (P < 0.01) (shown in **Table 3**), and no significant collinearity was detected among them by collinearity statistics. After adjusting for all the aforementioned risk factors using multivariate linear regression, HP infection was still identified as an independent factor associated with total skeletal muscle mass (β = -1.03, 95% CI = -1.31 to -0.75, P < 0.01).

**Table 3:** Influence of helicobacter pylori infection on total skeletal muscle mass.

Variables	Univariate analyse		Multivariate analyse	
	β (95%CI)	P	β (95%CI)	P
HP+	-3.12 (-3.51–2.79)	<0.01	-1.03 (-1.31–0.75)	**<0.01**
Clinical database				
Age (years)	-0.11 (-0.12–0.09)	<0.01	-0.11 (-0.12–0.09)	**<0.01**
BMI (kg/m^2^)	0.86 (0.81-0.91)	<0.01	0.60 (0.55-0.65)	**<0.01**
SBP (mmHg)	0.05 (0.04-0.06)	<0.01	-0.01 (-0.02-0.01)	0.86
DBP (mmHg)	0.09 (0.07-0.11)	<0.01	0.01 (-0.02-0.02)	0.65
Plasmea inflammatory markers				
WBC (10^9/L)	0.51 (0.39-0.62)	<0.01	-0.06 (-0.18-0.06)	0.31
hs-CRP (mg/L)	0.19 (0.11-0.26)	<0.01	-0.06 (-0.12-0.01)	0.08
NLR ratio	-0.02 (-0.02–0.01)	<0.01	-0.45 (-0.69-0.20)	**<0.01**
PLR ratio	-0.01 (-0.01–0.00)	<0.01	-0.01 (-0.01-0.00)	0.22
MLR ratio	3.06 (0.03-6.09)	<0.01	5.20 (0.05-10.45)	0.05
NMR ratio	-0.21 (-0.32—0.11)	<0.01	-0.15 (-0.31-0.00)	0.06
Nutrition-related index				
Albumin (g/L)	0.32 (0.25-0.39)	<0.01	-0.01 (-0.06-0.06)	0.91
Total protein (g/L)	-0.03 (-0.07-0.02)	0.27		
Hemoglobin (g/L)	0.19 (0.18-0.20)	<0.01	0.09 (0.08-0.10)	<0.01
Metabolic indexes				
FBG (mmol/L)	0.13 (0.02-0.25)	0.03	0.14 (0.05-0.23)	**<0.01**
TC (mmol/L)	-0.01 (-0.20-0.18)	0.93		
TG (mmol/L)	0.86 (0.74-0.97)	<0.01	0.07 (-0.04-0.18)	0.20
LDL-C (mmol/L)	0.28 (-0.06-0.51)	0.02	-0.33 (-0.51–0.14)	**<0.01**
HDL-C (mmol/L)	-7.45 (-8.02–6.87)	<0.01	-1.11 (-1.63–0.59)	**<0.01**
Creatinine (umol/L)	0.17 (0.16-0.18)	<0.01	0.11 (0.09-0.12)	**<0.01**
Uric acid (umol/L)	0.03 (0.02-0.03)	<0.01	0.01 (0.00-0.01)	**<0.01**

Similar to **Table 2**.

The bolded values represent statistically significant results.

### 3.5 Influence of HP infection on ASMI

According to the results of the univariate linear regression analysis (shown in **Table 4**), age, BMI, blood pressure, serum inflammatory markers (excepting NLR), nutrition-related parameters (excluding total protein), and metabolic markers (excluding TC) were all found to be associated with ASMI (P < 0.01), and no significant collinearity was detected among them. Consequently, all these variables were included in the multivariate linear regression analysis, and HP infection remained an independent factor associated with ASMI (β = -0.09, 95% CI = -0.14 to 0.06, P < 0.01).

**Table 4:** Influence of helicobacter pylori infection on appendicular skeletal muscle mass index.

Variables	Univariate analyse		Multivariate analyse	
	β (95%CI)	P	β (95%CI)	P
HP+	-0.50 (-0.58–0.42)	**<0.01**	-0.09 (-0.14–0.06)	**<0.01**
Clinical database				
Age (years)	-0.01 (-0.01–0.00)	**<0.01**	-0.01 (-0.01–0.01)	**<0.01**
BMI (kg/m^2^)	0.21 (0.20-0.22)	**<0.01**	0.18 (0.17-0.19)	**<0.01**
SBP (mmHg)	0.01 (0.01-0.01)	**<0.01**	0.01 (-0.01-0.01)	0.84
DBP (mmHg)	0.02 (0.02-0.03)	**<0.01**	0.01 (-0.01-0.01)	0.79
Plasmea inflammatory markers				
WBC (10^9/L)	0.09 (0.07-0.11)	**<0.01**	-0.02 (-0.04–0.01)	**0.01**
hs-CRP (mg/L)	0.05 (0.03-0.06)	**<0.01**	-0.01 (-0.02–0.01)	**0.01**
NLR ratio	-0.02 (-0.06–0.03)	0.46		
PLR ratio	-0.00 (-0.00–0.00)	**<0.01**	0.00 (-0.01-0.00)	**0.01**
MLR ratio	0.68 (0.03-1.34)	**<0.01**	0.53 (-0.20-0.19)	0.15
NMR ratio	-0.04 (-0.06–0.02)	**<0.01**	-0.01 (-0.04-0.01)	0.32
Nutrition-related index				
Albumin (g/L)	0.03 (0.02-0.05)	**<0.01**	0.01 (0.01-0.02)	**0.02**
Total protein (g/L)	-0.01 (-0.02-0.01)	0.22		
Hemoglobin (g/L)	0.03 (0.02-0.03)	**<0.01**	0.01 (0.01–0.01)	**<0.01**
Metabolic indexes				
FBG (mmol/L)	0.05 (0.03-0.08)	**<0.01**	0.02 (0.01-0.03)	**<0.01**
TC (mmol/L)	0.02 (-0.02-0.06)	0.31		
TG (mmol/L)	0.16 (0.13-0.18)	**<0.01**	-0.01 (-0.02-0.02)	0.95
LDL-C (mmol/L)	0.07 (0.02-0.11)	**0.01**	-0.04 (-0.07–0.01)	**0.01**
HDL-C (mmol/L)	-1.28 (-1.40–1.15)	**<0.01**	-0.12 (-0.20–0.04)	**<0.01**
Creatinine (umol/L)	0.02 (0.02-0.03)	**<0.01**	0.01 (0.01-0.01)	**<0.01**
Uric acid (umol/L)	0.00 (0.00-0.01)	**<0.01**	0.01 (0.01-0.02)	**<0.01**

Similar to **Table 2**.

The bolded values represent statistically significant results.

The subjects were further divided into young(n=3320), middle-aged(n=4324), and elderly(n=466) groups based on their ages. Consistent with our previous preliminary observation, individuals in the HP+ group had significantly lower total muscle mass, total skeletal muscle mass, and ASMI compared to the HP- group according to all age groups (all P < 0.05); after adjusting for all the aforementioned risk factors by multivariate linear regression, HP infection remained an independent factor affecting total muscle mass, total skeletal muscle mass, and ASMI in all age groups (all P < 0.05) (**Supplementary Figure 1**; **Table 1**).

### 3.6 Longitudinal association between HP infection status and different types of muscle mass

**Figure 2:**
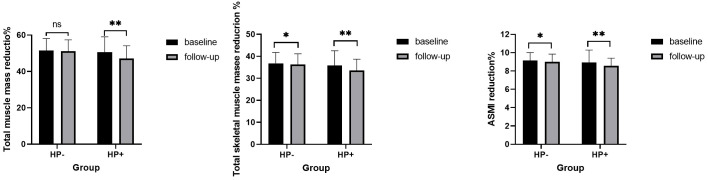
Comparison of different types of muscle mass during baseline and follow-up in subjects with and without helicobacter pylori infection. Similar to **Figure 1**. ns: P>0.05, * P<0.05; ** P<0.01.

A cohort of 426 individuals who underwent at least two medical examinations, with a time interval of more than 1 year between the initial and final check-ups, did not receive treatment for HP eradication, and maintained a consistent HP status throughout the follow-up period, was used to investigate the longitudinal association between HP infection and muscle mass (shown in **Table 5**). During the follow-up period, both total skeletal muscle mass and ASMI showed a significant reduction in both the HP+ and HP-groups (P < 0.05). Similarly, total muscle mass decreased during the follow-up period but only showed a significant reduction in the HP+ group (P < 0.05). The mean percentage reduction of total muscle mass, total skeletal muscle mass, and ASMI in the HP+ group were significantly higher than that of the HP- group (shown in **Figure 2**, all with all P < 0.01).

**Table 5:** Comparison of the percentage reduction in different muscle mass between subjects with and without helicobacter pylori infection.

Variable	HP-(n=221)	HP+(n=205)	P
Percentage of total muscle mass reduction	0.17(-2.30-1.85)	2.80(0.21-9.79)	**<0.01**
Percentage of total skeletal muscle mass reduction	0.28(-2.15-1.93)	2.79(0.14-10.26)	**<0.01**
Percentage of ASMI reduction	0.34(-1.33-0.34)	2.19(-1.14-6.69)	**<0.01**

ASMI, Appendicular skeletal muscle mass index; HP, Helicobacter pylori.

The bolded values represent statistically significant results.

## 4 Discussion

The present large cross-sectional and short study demonstrated that subjects with HP infection had lower muscle mass, total skeletal muscle mass, and ASMI as measured by BIA compared with those without HP infection. HP infection was an independent risk factor related to muscle mass, total skeletal muscle mass, and ASMI in adult Chinese males. The association persisted when the subjects were stratified into different groups based on their ages. Furthermore, subsequent cohort studies have demonstrated that continuing HP infection is associated with a notable reduction in muscle mass among individuals, indicating that HP infection may accelerate the pathological progression of muscle atrophy, independent of aging and traditional risk factors.

The underlying mechanism between HP infection and muscle atrophy may be associated with systemic chronic inflammation, malnutrition and metabolic disorders. First, HP infection is accompanied by the increasing production of various inflammatory factors, such as WBC count, hs-CRP, tumor necrosis factor-alpha (TNFα) and interleukin-6 (IL-6), interleukin-1β(IL-1β), interleukin-8, and interleukin-17 (Chen et al., 2022; Della Bella et al., 2023; Jiao et al., 2024). These inflammatory factors not only accelerate muscle protein degradation by upregulating the expression of muscle growth inhibitors (such as myostatin) and muscle atrophy-related proteins (such as MuRF1 and Atrogin-1) but also directly lead to a reduction in muscle mass by inhibiting muscle protein synthesis (Wang et al., 2017). Reducing the levels of inflammatory factors such as TNFα, IL-1, and IL-6 can upregulate the expression of myogenic regulatory factors, including Myf5, Myf6, MyoD, and myogenin, by activating the AMPK/SIRT3/PGC1α pathway (Han et al., 2024). On the other hand, mitochondrial dysfunction and ubiquitin proteasome system imbalance are important molecular mechanisms involved in the occurrence and development of skeletal muscle atrophy, studies have reported that H. pylori primarily uses its CagA protein to increase the level of mitophagy, block autophagic flux, cause mitochondrial dysfunction and ubiquitin-proteasome system imbalance by regulating the activation of the NLRP3 inflammasome (Coombs et al., 2011; Chen D. et al., 2024; Ji et al., 2025). Additionally, suppressing oxidative stress and inflammation can reverse the pathological progression of skeletal muscle atrophy (Chang et al., 2025). In the present study, some inflammatory indicators, including hs-CRP, NLR, NMR, and MLR, were higher in the HP+ group compared with the HP- group, and WBC, hs-CRP, PLR, MLR, and NMR were all related to total muscle mass, total skeletal muscle mass, and ASMI in univariate linear regression analysis.

Second, HP infection not only often leads to restricted absorption of various nutrients such as proteins, fats, and carbohydrates but also severely affects the absorption of certain micronutrient, such as vitamin D,vitamin B12 and iron (Franceschi et al., 2014; Zhang et al., 2024). A protein–energy deficit can reduce muscle synthesis, quickly lead to a loss of muscle mass, strength, and function (Sloane et al., 2019). Additionally, a deficiency of vitamin D will directly inhibit the proliferation and differentiation of myoblasts, leading to a decrease in the repair capacity of muscle tissue (Romeu Montenegro et al., 2019). Furthermore, an HP infection can affect vitamin B12 absorption, a crucial coenzyme in the mitochondrial methylation cycle,which can inhibit mitochondrial fatty acid oxidation, and its serum levels are closely related to the energy supply to muscles, being positively correlated with muscle strength and mass (Gillies et al., 2021; Li et al., 2021; Zhao et al., 2024). Finally, Helicobacter pylori infection reduces gastric and ascorbic acid levels by inhibiting the transcriptional activity of HK-ATPase in parietal cells (Schubert, 2008), which can interfere with the absorption of iron. A thorough literature review was conducted using the PubMed/MEDLINE/Web of Science databases, indicating that subjects with HP infection are more likely to develop iron deficiency anemia (IDA), combining HP eradication with iron supplementation is more effective in treating IDA (Pu et al., 2025), this is consistent with the study’s finding that 69.4% of anemic patients were infected with HP. In the present study, the serum levels of ALB, TP, HGB, TG, LDL-C, creatinine, and uric acid, which can reflect nutrition and metabolic conditions in the HP+ group, were all found to be lower than those in the HP- group (P < 0.05) and were further identified as independent influencing factors for total muscle mass, total skeletal muscle mass, and ASMI by univariate and multivariate linear regression analysis.

In addition, changes in the secretion of some hormones caused by HP infection may be another mechanism related to muscular atrophy. Studies have demonstrated that HP infection decreases the serum levels of estradiol and ghrelin in both male and female subjects (Gennari et al., 2021). Decreased estradiol has been implicated in the accompanying loss of skeletal muscle mass and strength due to its influence on skeletal muscle mitochondrial dysfunction (Moreira-Pais et al., 2024). Ghrelin has been proposed as a treatment for muscle atrophy because it can directly increase muscle mass by increasing appetite and indirectly promoting myocyte differentiation and fusion by activating its receptor (Filigheddu et al., 2007; Kim et al., 2024).

### 4.1 Limitations

This study also has certain limitations. First, as a retrospective study, it failed to fully demonstrate the dynamic impact of HP eradication on the process of muscle decline in males, indeed, Baeg et al. (Baeg et al., 2015) have illustrated that elderly women who received HP-eradicating therapy have a reduced risk of low skeletal muscle mass. Further research will be conducted to validate the impact of HP eradication on the progress of muscle atrophy in males. Second, this study did not collect indicators of muscle strength and function. Thus, it could not fully assess the potential impact of HP infection on muscle health. More detailed variables will be introduced to investigate this aspect in future studies to accurately reveal the impact of HP infection on muscle health. Thirdly, the potential limitations of BIA assessment depend on various factors, including body moisture, body temperature, and food intake. Nonetheless, the subjects in the present study were instructed to fast and strictly adhere to the guidelines for BIA inspection (Kyle et al., 2004). Finally, the present study has only found a correlation between body muscle mass and HP infection, the underlying mechanism still requires further study.

## 5 Conclusion

The present study confirmed that subjects with HP infection had lower total muscle mass, total skeletal muscle mass, and ASMI compared to those without HP infection. Thus, HP infection was an independent risk factor related to muscle decline in adult Chinese males, regardless of different ages and traditional risk factors. This finding suggests that treatment for HP eradication may be recommended to prevent muscle decline.

## Data Availability

The raw data supporting the conclusions of this article will be made available by the authors, without undue reservation.

## Supplementary material

Supplementary Figure 1 Comparison of different types of muscle mass in subjects with and without helicobacter pylori infection according to different age groups. ASMI, Appendicular skeletal muscle mass index; HP, Helicobacter pylori. ^*^<0.05. ^**^<0.01.

Supplementary Table 1 Influence of helicobacter pylori infection on different types of muscle mass according to age groups.
