# Supplementary figures and images for: Influence of helicobacter pylori infection on Chinese adult males’ body muscle mass: a cross-sectional and cohort analysis
Source: Front Cell Infect Microbiol. 2025 May 29;15:1575108. doi: 10.3389/fcimb.2025.1575108 (PMC12159044; doi:10.3389/fcimb.2025.1575108)

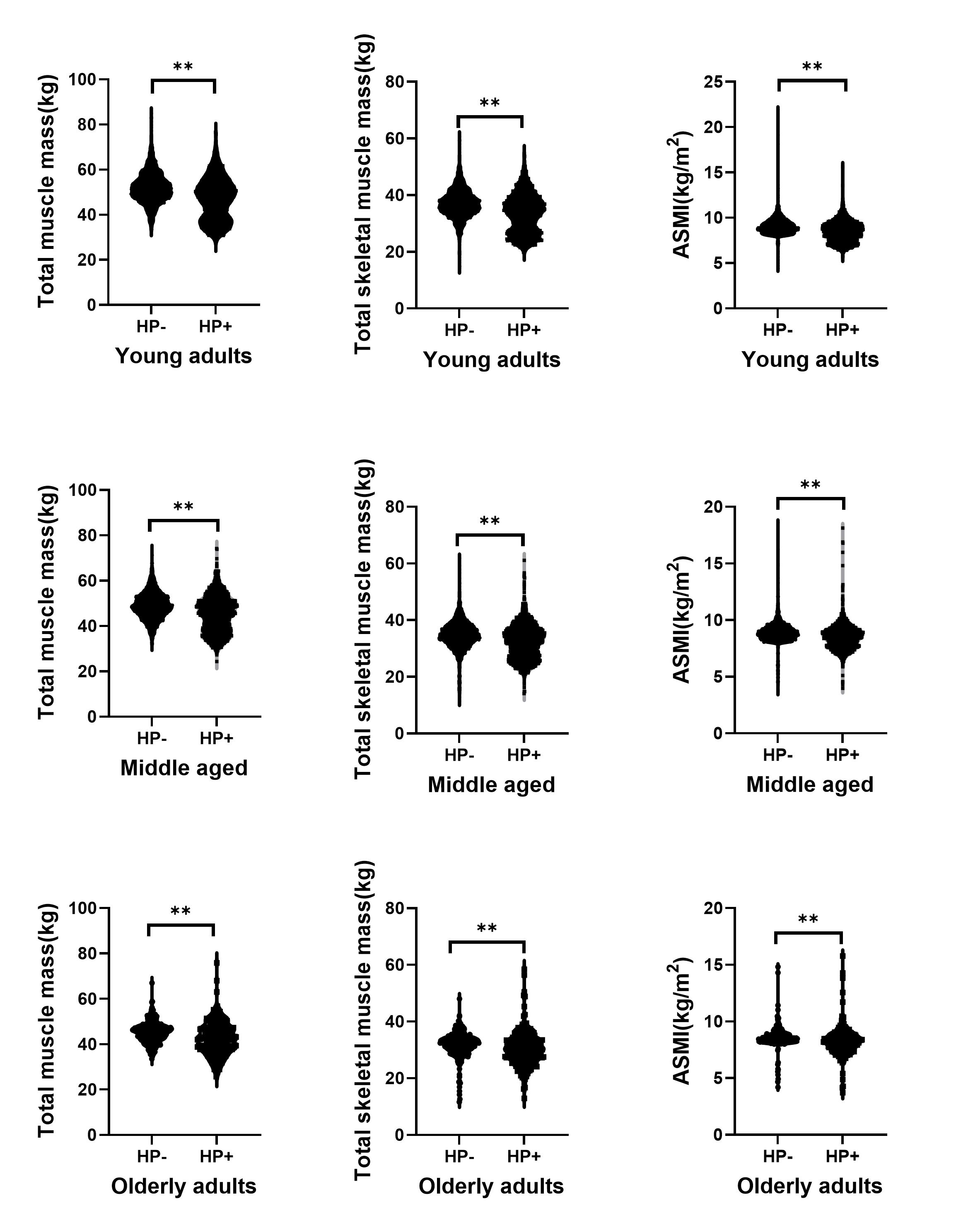

Supplement: Supplementary Figure 1 — Comparison of different types of muscle mass in subjects with and without helicobacter pylori infection according to different age groups. ASMI, Appendicular skeletal muscle mass index; HP, Helicobacter pylori. *<0.05. **<0.01. [file Image1.jpeg]
