# Supplementary material for: Influence of helicobacter pylori infection on Chinese adult males’ body muscle mass: a cross-sectional and cohort analysis
Source: Front Cell Infect Microbiol. 2025 May 29;15:1575108. doi: 10.3389/fcimb.2025.1575108 (PMC12159044; doi:10.3389/fcimb.2025.1575108)
Supplement: Supplementary Table 1 — Influence of helicobacter pylori infection on different types of muscle mass according to age groups. [file Table1.docx]

**Supplementary table 1.**Influence of helicobacter pylori infection on different types of muscle mass according to age groups

| Variable | β | 95%CI | P |
| --- | --- | --- | --- |
| **Young adults (age below 45years，n=3320)** | | | |
| Total muscle mass^*^ | -1.83 | -2.45--1.91 | <0.01 |
| Total skeletal muscle mass^*^ | -1.11 | -1.58--0.64 | <0.01 |
| ASMI^$^ | -0.09 | -0.15--0.04 | <0.01 |
| **Middle-aged (age between 45 and 60 years.n=4324)** | | | |
| Total muscle mass^*^ | -1.72 | -2.18--1.26 | <0.01 |
| Total skeletal muscle mass^*^ | -0.91 | -1.28--0.54 | <0.01 |
| ASMI^#^ | -0.07 | -0.13--0.02 | 0.01 |
| **Elderly adults (age above 60 years.n=466)** | | | |
| Total muscle mass^*^ | -1.31 | -2.54--0.08 | 0.04 |
| Total skeletal muscle mass^*^ | -1.46 | -2.53--0.39 | 0.01 |
| ASMI^#^ | -0.25 | -0.46--0.04 | 0.02 |

**Abbreviations:**ASMI, appendicular skeletal muscle mass index.

* Adjusted for age, BMI, SBP, DBP, WBC, hs-CRP, NLR, MLR, NMR, albumin, hemoglobin, FBG, TG, LDL-C, HDL-C, creatinine, and uric acid (similar to Table 2 and Table 3).

^#^ Adjusted for age, BMI, SBP, DBP, WBC, hs-CRP, MLR, NMR, albumin, hemoglobin, FBG, TG, LDL-C, HDL-C, creatinine, and uric acid (similar to Table 4).
